# Application of Bayesian Approach in Cancer Clinical Trial

**DOI:** 10.14740/wjon842e

**Published:** 2014-06-25

**Authors:** Atanu Bhattacharjee

**Affiliations:** Division of Clinical Research and Biostatistics, Malabar Cancer Center, Thalassery, Kerala 670103, India. Email: atanustat@gmail.com

**Keywords:** Markov chain Monte Carlo, Iteration, HPD and likelihood

## Abstract

The application of Bayesian approach in clinical trials becomes more useful over classical method. It is beneficial from design to analysis phase. The straight forward statement is possible to obtain through Bayesian about the drug treatment effect. Complex computational problems are simple to handle with Bayesian techniques. The technique is only feasible to performing presence of prior information of the data. The inference is possible to establish through posterior estimates. However, some limitations are present in this method. The objective of this work was to explore the several merits and demerits of Bayesian approach in cancer research. The review of the technique will be helpful for the clinical researcher involved in the oncology to explore the limitation and power of Bayesian techniques.

## Introduction

Bayesian approach is an emerging technique in medical research. Public health research to clinical trials is now becoming dependent on Bayesian. Several clinical trials are now running with design of Bayesian approach. The drug treatment comparison with Bayesian is now approved with FDA. However, the procedure for analyzing and interpretation of data is different in Bayesian approach in comparison to classical. The computational advantage and complex issue in data analysis give the up-gradation of Bayesian approach over classical methods. The field of oncology is involved with case fatality. The survival analysis is one of the complex issues in any clinical trial. Usually, the clinical trials are affected with dropout’s observations. The dropout of observations can be estimated in presence of observed observation. Bayesian plays a crucial role in estimations of observed data and draws influence about it. The growth rate of applying Bayesian in clinical trial is tremendous. The footprint can be captured through medical journals and clinical trial projects. Recently, the oncology trial result through Bayesian approach has been published [1]. The Bayesian study design is adopted for the cancer clinical trial conducted in several renowned cancers institutes in the world. In this connection, the US FDA has also accepted the Bayesian design for any clinical trial. The Bayesian probability is something different than conceptual probability [2]. It is based on evidence-based probability. It gives the extension on the propositional logic to enable the proposition about truth and false of the uncertainty. The specific prior is important to formulate the hypothesis. However, the literature review is important to obtain the prior information. It is also widely accepted as scientific tools in other research area like cancer genetic [3-6], systematic review and policy/decision [7]. The aim of the paper was to elaborate the Bayesian approach to the cancer researcher. The broad goal was to provide a platform to the scientific community to accept the Bayesian approach for clinical trial.

## About Bayesian

The Bayesian is based on coherent learning from the experimental data. It is an inductive inferential approach through consideration of observed outcome and enlarging it to the uncertainty in the general population. In contrast to the Bayesian, the classical approach is deductive procedure for concluding the inference about the population parameters. The Bayesian is open to cop the information from multiple sources and draw the inference about the population concerned.

The way to draw inference and merge evidence through Bayesian is something different than classical approach. It is also useful to merge the evidence from different sources to provide the output. The inductive approach is considered in the Bayesian from the observed outcome, expanding the statement for the general population. The deductive procedure is applicable to the classical approach only.

## The Procedures in Bayesian Approach

Three points are important in Bayesian approach, i.e. 1) distribution of the prior; 2) likelihood to generate the posterior; and 3) distribution of the posterior. The relation between posterior, prior and likelihood is posterior = likelihood × prior.

The relation and procedures can be elaborated with examples. Let the main objective was to determine the treatment effect to reduce the tumor growth in cancer patients. The experimental treatment is A and reference is B. Now the distribution information about effectiveness of treatment to reduce the tumor growth among patients with treatment A and treatment B are required. This particular information will be considered as prior information to generate posterior estimates. The next step is to formulate the experimental data as likelihood over the treatment B. Now in this step the prior information is multiplied with likelihood. The multiplied value will be the posterior estimates of the current study. Now the posterior estimate is useful to give any reply about treatment effect A.

## Prior Information

The important part of Bayesian approach is the prior information. It is the information available before the study starts. The information is to be generated through earlier performed study. The priors can be separated into informative or non-informative. The non-informative prior gives equal chances of all possible values to occur in the experiment. The result observed through informative prior is complete image of the data alone. The informative prior is selective to be included in the sample. There are different types of technique to create the data-based priors. Different types of priors are important to carry out the analysis. The prior can be classified as follows. 1) Conjugate prior: family of distribution of the prior and posterior is same. It can be further divided into informative and non-informative. 2) Non-informative prior: flat prior relative to the likelihood function is called non-informative prior. The impact of non-informative prior is minimal to decide the posterior. 3) Locally uniform prior: prior does change over the region in which the likelihood is appreciable, and does not assume large values outside that range. 4) Reference prior: this prior is convenient to use as a standard. 5) Location-invariant prior: prior that is invariant to the choice of the origin (e.g., like body temperature of the patients). 6) Scale-invariant prior: prior that is invariant to the scale of measurement (e.g., like inference on the height of the patients in CM). 7) Improper priors are often used because they generally yield non-informative priors.

## Contrasting Bayesian and Classical Statistics

Both of the approaches have their power and restraint in different prospects. The Bayesian and classical both can be altered to each other to serve specific objective. It is difficult to point best approach in any problem. Bayesian is built with concept of probability. The classical is based on experimental data to draw information about parameter of interest.

## Bayesian in Complex Problem

The Bayesian is simpler to solve raised complex problem in clinical trial. It provides more consistent frame to solve the research question to the counted information. The frame is commonly applicable for simple and complex problem. For instance, clinical trial becomes complicated due to occurrence of missing observation. The Bayesian is ultimate choice to solve the issue. Basically, three steps are important to work with Bayesian, i.e. 1) specifying the prior distribution of the parameter of interest; 2) observing the data of interest; and 3) formulation of posterior distribution through the assistance of likelihood and prior information. Pattern mixture modeling is one of the best methods to handle missing observation through Bayesian approach [8].

## Bayesian Clinical Trial Decision

Bayesian gives the platform to monitor the trial in terms of “utility” and “loss”. In cancer clinical trial, the success of treatment can be measured by “utility” function. The development of toxicity due to treatment can be captured through “loss” value. The way of interpreting data from different investigators may be different like cured, suffering and occurrence of toxicity. But the Bayesian addresses the issue through informed decision of “utility” and “loss” functions.

## Application of Bayesian Model in Clinical Trials

Open source software like WinBUGS and R is useful for Bayesian data modeling. The important statistical methods for clinical trial data analysis are survival and longitudinal. The packages available in R are suitable clinical trial data analysis. The Open Bugs package available in R is compatible to work with WinBUGS and R. The important part to work with Bayesian approach is Markov chain Monte Carlo (MCMC) procedure. The MCMC can be carried out for data analysis in both the packages. The MCMC is basically the simulation-based approach to draw inference about posterior means. It is equally effective in longitudinal and survival data analysis [3, 8, 9].

## Bayesian Stopping Rule in Clinical Trial

Clinical trial can be conducted through retrospective and prospective manners. In case of prospective trial the stopping rule is important for study continuation. Based on the available information of the trial, the decision about study continuation can be considered. The estimates of the likely success can be obtained through Bayesian. The dynamic approach of stopping the trial can save time, money and unnecessary exposure with toxic drug to the patients. The Bayesian is more useful and simple to apply on the current posterior distribution value. The approach can be considered for data monitoring tool as well.

## Bayesian in Sequential Design

The main objective in any clinical trial was to determine the patient’s survival. The standard sequential approach assumed the treatment having event distribution with proportional hazard model. The stopping/conducting rule about the trial is well-documented [10]. The level of stopping boundaries is decided through false-positive and power value. During the interim analysis, the log-rank value is estimated based on current data and compared for decision about trial to stop or continue. The Bayesian adaptive method is fit for the sequential trial. It requires less number of sample sizes. The sample size to stop/complete data can be decided based on the current data in hand.

## Bayesian With Predictive Probability

Sequential analysis is more useful through Bayesian. In sequential analysis, the updated information is used to generate further information, and the processes keep on carrying. In Bayesian, the prior gives the scope to obtain posterior information. The obtained posterior becomes used as prior for next posterior generation. The sequential analysis merged with Bayesian gives more consistent results in comparison to frequentist method. Sequential analysis is ultimate choice for any trial monitoring. The trial monitoring becomes useful to protect patient from toxic treatment effect. The frequentist approach is based on the assumption through the fixed value of the parameter of interest to generate the decision about analysis [11]. In contrast, Bayesian assumes the random effect of the parameter of interest to generate the conclusion about the trial. The fixed value of the parameter of interest may be biased, and formulate the biased result. Whereas, the chances of getting the biased result due to consideration of random effect of the parameter of interest are certainly low in Bayesian [11].

## Bayesian in Adaptive Study Design

The adaptive design is useful for collecting data to take decision and modify the features to study without offering the validity and integrity of the data. It involves the continuous procedure for decision taking. It is useful to assign the stopping rule and terminate the study immediately due to occurrence of any serious adverse event. The Bayesian is suitable to handle with adaptive study design.

## Bayesian in Meta Analysis

Meta analysis is pooled analysis of information generated from different studies. It is natural that different studies will differ from characteristics of the patients, size of trial, study design, methodology and cohort. The components are required to be addressed through pooled analysis of the data. The characters can be assumed to be random from trial to trial. The random effect is the only choice to solve the problem. The Bayesian is best fitted approach to deal with random effects [11, 12]. Bayesian puts the scope to draw conclusion of the unanswered questions in controversial clinical trial. It is better fitted for decision analysis on the cost-effective studies. Comprehensive example of Bayesian meta analysis is available [13, 14].

## Limitation of Bayesian

The most discussed area with Bayesian is the selection of prior. Frequentist criticized the application of Bayesian due to presence of prior selection. The selection of prior sometimes becomes selective and biased. The wrongly selected prior can influence the false inference about the objective. It should be cautious for selection of prior. The prior should not influence the generation of the posterior distribution. It is natural that the posterior will be generated through prior information. But the influence on the posterior of the prior should be zero. However, the problem can be sorted by selection of non-informative and flat prior. The conjugate prior also gives good results for posterior generation. If researcher is interested in generating posterior from the desired family, then it is best to choose the conjugate prior. The conjugate prior provides the posterior from the same family of distribution. Although the Bayesian has more potential to solve problems in clinical trial and to be applied in broad spectrum, it is not widely accepted due to presence of computation difficulties. The reason may be that researchers are not aware of the methods. The article is contributed to explore the advantages and disadvantages of Bayesian in cancer clinical trial. Any prospective and retrospective cancer clinical trial can be conducted through Bayesian approach.

The required steps to be incorporated for Bayesian approach are detailed by Sung and his colleagues [15]. The points are: 1) brief description of prior and distribution of the prior; 2) detailed justification about the prior that has been to be chosen; 3) proper elaboration of the statistical model to fulfill the study objectives; and 4) the detail about posterior estimate and standard deviation with 95% credible interval.

## Conclusion

The best idea is the conjoint work with classical and Bayesian approach. There are certain areas, where classical approach is inevitable and some areas for Bayesian. The dose-level monitoring, sample size determination and complex issues raised due to study design are advised to be tackled with Bayesian approach. The simple drug treatment exploration, exploratory data analysis and logistics approach can be addressed by classical approach. Therefore, Bayesian is in tremendous growth in terms of acceptance in medical research. The sole quality of the Bayesian attracts the researcher to apply Bayesian in clinical trial.
